# Opioid Agonist Therapies and Pregnancy Outcomes for Pregnant People With Opioid Use Disorder: Protocol for a Systematic Review

**DOI:** 10.2196/42417

**Published:** 2023-05-10

**Authors:** Lindsay A Wilson, Preet Gandhi

**Affiliations:** 1 Faculty of Medicine University of British Columbia Vancouver, BC Canada

**Keywords:** opioid agonist therapies, opioid use disorder, pregnancy, opioid, drug, symptoms, treatment, screening, data, risk, clinical, policy makers, community

## Abstract

**Background:**

Opioid use disorder (OUD) during pregnancy presents a significant risk to maternal, fetal, and neonatal health, increasing the likelihood of adverse events, such as maternal overdose, pregnancy loss, stillbirth, preterm birth, low birth weight, and neonatal abstinence syndrome. In order to reduce the risk of these outcomes, the standard of care for OUD during pregnancy in many jurisdictions within the United States and Canada is opioid agonist therapy (OAT). OAT refers to prescription medications that alleviate or eliminate opioid withdrawal symptoms, so that opioid use can be managed more safely. Although OAT has been recognized as a safe option for pregnant people with OUD, many jurisdictions do not have treatment guidelines regarding pharmacological options, dosing recommendations, side effect management, and individual preferences. There is currently a lack of systematic evidence on the impacts of different OAT regimens on pregnancy outcomes.

**Objective:**

We aim to evaluate the impacts of specific OAT agents on pregnancy outcomes and inform recommendations for practitioners treating pregnant people with OUD.

**Methods:**

The MEDLINE, Embase, CINAHL, and PsycINFO databases will be searched for published quantitative studies assessing pregnancy outcomes for individuals on OAT. Given the substantially increased risk of preterm birth, low birth weight, small for gestational age, and stillbirth among pregnant people with OUD, these four end points will comprise our primary outcomes. Database searches will not be restricted by date, and conference abstracts will be restricted to the past 2 years. Titles, abstracts, and full-text articles will be independently screened by 2 reviewers. Data will be extracted independently and in duplicate, using a data extraction form to reduce the risk of reviewer bias. The risk of bias within individual studies will be assessed by using the appropriate CASP (Critical Appraisal Skills Programme) checklists. For studies that consider the same research questions, interventions, or outcomes, meta-analyses will be conducted to synthesize the pooled effect size. In the event that studies cannot be compared directly, results will be synthesized in a narrative account. Between-study heterogeneity will be measured by using the τ^2^ statistic. If more than 10 studies are available for pooling, publication bias will be evaluated by using the Egger regression test.

**Results:**

As of January 2023, a total of 3266 abstracts have been identified for screening. Data extraction is expected to commence in February 2023.

**Conclusions:**

The topic of OAT and its effect on pregnancy is an understudied area that has the potential to improve health outcomes, clinical practice, education, and community advocacy. The results of our review will be used to inform clinical practice guidelines and improve health outcomes for pregnant people. Findings will be disseminated to diverse groups of stakeholders, including policy makers, clinicians, community partners, and individuals with lived experience of drug use.

**Trial Registration:**

PROSPERO CRD42022332082; https://tinyurl.com/2p94pkx5

**International Registered Report Identifier (IRRID):**

DERR1-10.2196/42417

## Introduction

Opioid use disorder (OUD) during pregnancy presents a significant risk to maternal, fetal, and neonatal health, increasing the likelihood of adverse events, such as maternal overdose, pregnancy loss, stillbirth, preterm birth, low birth weight, and neonatal abstinence syndrome [1,2]. Although OUD during pregnancy is associated with considerable harms, the withdrawal symptoms associated with sudden cessation are also extremely harmful to both the person who is pregnant and the fetus [3,4].

In order to reduce the risk of these outcomes, the standard of care for OUD during pregnancy in many jurisdictions within the United States and Canada is opioid agonist therapy (OAT) [3]. OATs are prescription medications that alleviate or eliminate opioid withdrawal symptoms so that OUD can be managed more safely. Buprenorphine and methadone are both recommended as first-line treatments in many jurisdictions, with buprenorphine increasingly being recommended as the preferred therapeutic option, given its established safety profile and simple treatment regimen [3]. Other options, such as slow-release oral morphine, can also be used for individuals who do not respond to first- or second-line therapies [3]. The ongoing use of OAT throughout pregnancy is considered safe for both the person who is pregnant and the fetus [3-5].

Although OAT offers a relatively safe option for managing OUD during pregnancy, many health care providers are unfamiliar with how best to prescribe OAT throughout a pregnancy, and many jurisdictions do not have established treatment guidelines. A consistent dosage can be difficult to maintain as the body changes during pregnancy, putting the person who is pregnant at risk of experiencing withdrawal symptoms or overdose both during the pregnancy and post partum [6-8]. These limitations, in combination with the fact that OAT can produce side effects (eg, nausea, vomiting, and constipation) [9] and that some pregnant people may simply prefer to continue using opioids, can increase the complexity of treatment regimens and approaches for ensuring that pregnant people receive prenatal care that is the most appropriate for them.

Although there is considerable literature to suggest that OAT is preferable to opioid use during pregnancy, to our knowledge only 1 systematic review has been conducted that considers the impacts of OAT on pregnancy outcomes. This living systematic review, which was conducted by Minozzi and colleagues [10], offers the most up-to-date evidence from randomized controlled trials (RCTs) to support the use of OAT during pregnancy. However, the authors note that the small number of RCTs considering this topic (n=4) makes it difficult to draw firm conclusions about the relative benefits of different OAT regimens, and further research is still needed [10]. Given the limited number of studies and participants available for inclusion from RCTs, we sought to build upon the existing knowledge base by evaluating observational studies that have been conducted to evaluate impacts of various types of OAT on pregnancy outcomes. Although observational studies include greater bias and heterogeneity when compared to RCTs, the inclusion of additional studies containing larger numbers of participants can offer valuable insights into the real-world impacts of OAT on pregnancy outcomes.

## Methods

### Protocol and Registration

This systematic review protocol follows the PRISMA-P (Preferred Reporting Items for Systematic Review and Meta-Analysis Protocols) checklist [11] and has been registered in the PROPSERO database (ID number: CRD42022332082). The full systematic review will also follow the PRISMA (Preferred Reporting Items for Systematic Reviews and Meta-Analyses) guidelines.

### Research Question

The systematic review will seek to address the question “What impact do different forms of OAT have on pregnancy outcomes among pregnant people with opioid use disorder?”

### Eligibility Criteria

The population of interest in our review will be pregnant people with OUD. The exposure will be OAT (eg, methadone, buprenorphine/naloxone [Suboxone], slow-release oral morphine, hydromorphone, and naltrexone). The primary outcomes of interest for the review will be preterm birth (ie, gestational age<37 weeks), low birth weight (ie, <2500 g), small for gestational age birth weight (ie, birth weight<10th percentile for gestational age), and stillbirth. Although many other maternal and infant health outcomes are impacted by OAT, we have opted to limit the scale of our results by restricting our analysis to these four outcomes, given the substantially increased risk of these outcomes among people with OUD [12-14] and the widespread availability and uniformity of the data that have been collected on these outcomes, which will facilitate comparisons between studies. Study eligibility criteria are presented in Table 1. Our planned start date for data extraction is February 1, 2023.

**Table 1:** Study eligibility criteria.

	Inclusion criteria	Exclusion criteria
Article type	Published studies Randomized clinical trials Case-control studies Cohort studies Cross-sectional studies Conference abstracts published within the previous 2 years	Unpublished or grey literature Case studies Case series Editorials Opinion pieces Commentaries Conference abstracts older than 2 years
Study design	Inclusion of individuals who engage in illicit opioid use Comparison of one form of opioid agonist therapy to another or to no intervention	Inclusion of only individuals who engage in other forms (ie, nonopioid) of drug use No comparator group
Language	English	All other languages
Date Range	No restrictions	None
Setting	No restrictions	None

### Information Sources and Search Strategy

We will search MEDLINE, Embase, CINAHL, and PsycINFO to identify studies relevant to our review. Search terms are aligned with the eligibility criteria described above, including variations on “opioid agonist therapy,” “pregnancy,” “opioid use,” and each of the pregnancy outcomes of interest. These terms were used as keywords and mapped onto Medical Subject Headings terms. Our search strategy was developed in consultation with a medical librarian at the University of British Columbia to ensure that there were no major omissions in our approach (the MEDLINE search strategy is presented in Textbox 1).

**Search strategy for Embase.**

**Searches**

1. *exp Buprenorphine/ or Opiate Substitution Treatment*
2. *Methadone/*
3. ((methadone or methadose or “opioid agonist therap*’” or “opioid agonist treatment*” or “opiate agonist therap’” or “opiate agonist treatment* or “medication assisted treatment’” or “medication assisted therap*” or OAT or “medication assisted recovery” or “opioid replacement therap*” or buprenorphine or suboxone or “opiate treat*” or “opioid treat*” or “Injectable hydromorph*” or medication*) adj2 “opioid use disorder”).tw,kw.
4. Searches 1 *or* 2 *or* 3
5. (pregnan* or prenatal or perinatal or “birth weight” or birthweight or stillbirth or miscarriage or preterm birth or pre-term birth or remature* or “gestational age”).mp. [mp=title, abstract, heading word, drug trade name, original title, device manufacturer, drug manufacturer, device trade name, keyword heading word, floating subheading word, candidate term word]
6. Searches 4 *and* 5
7. Limit search 6 to English language
8. Limit search 7 to “humans only (removes records about animals)”
9. Limit search 8 to “remove preprint records”
10. Limit search 9 to conference abstracts
11. Limit search 10 to *yr=“2019-Current”*

### Study Selection and Data Extraction

From our database searches, we will remove any duplicate articles and then import all articles into Covidence (Veritas Health Innovation Ltd) [15]. Titles and abstracts will be reviewed in 1 stage, followed by a separate full-text screening of the remaining articles. During each stage, studies that do not meet the review criteria will be excluded. Each stage will be conducted by 2 investigators independently and in duplicate. Any disagreements will be resolved by consensus.

A data extraction form will be developed prior to article analysis. Data on study types, settings, sample sizes, types of OAT, primary and secondary outcomes, main study results, and recommendations will be extracted. Extraction will be conducted by 2 investigators independently and in duplicate and then reviewed collaboratively. Any additions that need to be made to the extraction form will be discussed between the two investigators.

### Risk of Bias

Given our inclusion of observational studies, which inherently contain some risk of bias, the risk of bias within individual studies will be assessed by using the CASP (Critical Appraisal Skills Programme) cohort study checklist and CASP case-control study checklist [16]. These checklists comprise 12 and 11 questions, respectively, that seek to analyze a study’s validity, results, and clinical utility; bias is assessed with regard to the exposure, outcome, study design, and analysis. Bias in each full-text study will be assessed by 2 reviewers independently using the CASP checklists. Disagreements will be resolved by consensus. Issues such as heterogeneity in outcome measures, OAT dosage, and reporting will be explored as potential issues.

### Data Synthesis

A PRISMA flowchart will be developed to indicate the number of articles identified and how many were excluded (including reasons for exclusion) at each stage of evaluation. For studies that consider the same research questions, interventions, or outcomes, meta-analyses will be conducted to quantitatively synthesize the pooled effect size of OAT on each outcome (ie, preterm birth, stillbirth, low birth weight, and small for gestational age) [17]. Subgroup analyses will be conducted by outcome and type of OAT regimen. However, given the likelihood of heterogeneity between studies, a meta-analysis may not be appropriate in all cases. In the event of studies with research designs, interventions, and outcomes that cannot be compared directly, the results of the data extraction will be synthesized in a narrative account [17,18]. Between-study heterogeneity will be measured by using the τ^2^ statistic, which captures the variance between studies and is better suited than other measures of heterogeneity to evaluate observational studies with large sample sizes [19]. If more than 10 studies are available for pooling, publication bias will be evaluated by using the Egger regression test [20]. If publication bias is detected, pooled estimates will be adjusted by using the trim and fill technique, which corrects for publication asymmetry [20,21].

## Results

A search strategy has been finalized with a medical librarian at the University of British Columbia. The initial search has yielded 3266 abstracts for screening. Data extraction is expected to commence in February 2023, and final results should be available within 6 months of the publication of this protocol.

## Discussion

Our proposed systematic review will be the first to synthesize observational data on the effect of different OAT options on pregnancy outcomes for people with OUD. The topic of OAT and its effect on pregnancy is an understudied area that has the potential to improve health outcomes, clinical practice, education, and community advocacy. The results of our review will primarily be used to inform clinical practice guidelines for OAT prescribing practices among pregnant people and subsequently improve health outcomes for both pregnant individuals and their infants. The results of our study will generate pertinent considerations for future research to continue improving the knowledge available on the effect of OAT on pregnancy.

This systematic review faces some limitations. There will be a degree of heterogeneity among the studies selected due to the variability of the research methodologies, settings, and OAT regimens and dosages being studied. We will evaluate this issue during data extraction and will adjust our analysis strategy accordingly. Further, in order to mitigate the risk of reviewer bias, we will have 2 reviewers independently screen and assess (ie, quality assessment) each study in our review. There is also a risk of missing pertinent literature on our research topic. We have consulted a medical librarian at the University of British Columbia and developed a broad search strategy to maximize the likelihood of identifying relevant articles. Lastly, publication bias is a general limitation of systematic reviews. We will attempt to mitigate this issue by including abstracts that have been published within the last 2 years, but we may miss some studies that did not reach the publication stage. We will adjust our conclusions based on evidence of publication bias as appropriate.

Our systematic review will synthesize relevant literature and integrate the vast observational data available on OAT and its impacts on adverse events during pregnancy. The findings of our study will contribute to improved clinical care and health for all pregnant people who use opioids for the duration of their pregnancies and beyond.

## Abbreviations

CASP: Critical Appraisal Skills Programme
OAT: opioid agonist therapy
OUD: opioid use disorder
PRISMA: Preferred Reporting Items for Systematic Reviews and Meta-Analyses
PRISMA-P: Preferred Reporting Items for Systematic Review and Meta-Analysis Protocols
RCT: randomized controlled trial

## Data Availability

Data sharing is not applicable to this article, as no data sets were generated or analyzed during the study.
